# Different drying methods drive divergent quality profiles in sea buckthorn protein hydrolysates: antioxidant preservation versus aroma complexity

**DOI:** 10.1016/j.fochx.2026.103566

**Published:** 2026-01-20

**Authors:** Chen Chen, Pan Zhang, Yuqian Wei, Xiaoyue Liang, Le Wang, Xizhe Fu, Jian Zhang, Yue Zhao

**Affiliations:** a Key Laboratory of Agricultural Product Processing and Quality Control of Specialty (Co-construction by Ministry and Province), School of Food Science and Technology, Shihezi University, Shihezi, Xinjiang 832000, China; b Key Laboratory for Food Nutrition and Safety Control of Xinjiang Production and Construction Corps, School of Food Science and Technology, Shihezi University, Shihezi, Xinjiang 832000, China; c Engineering Research Center of Storage and Processing of Xinjiang Characteristic Fruits and Vegetables, Ministry of Education, School of Food Science and Technology, Shihezi University, Shihezi, Xinjiang 832000, China

**Keywords:** Sea buckthorn protein hydrolysates, Drying methods, Antioxidant activity, Flavor characteristics

## Abstract

Drying critically impacts protein structure and function. Sea buckthorn peptide industrialization is challenged by preserving structural integrity and bioactivity during drying, as thermal and mechanical stresses alter protein properties and flavor. Systematic comparisons of drying methods for sea buckthorn protein hydrolysates are lacking. This study evaluated vacuum freeze-drying (VFD), Spray-drying (SD), and microwave vacuum drying (MVD) effects on sea buckthorn protein hydrolysates (SBP) structure, antioxidant capacity, and flavor. VFD-SBP demonstrates exceptional antioxidant properties (DPPH and ABTS radical scavenging rates of 72.34 ± 4.32% and 81.56 ± 2.99%), augmented amino acid retention capabilities, and enhanced thermal stability, respectively. SD-SBP possessed richer volatiles with a roasted nutty aroma, while VFD-SBP better preserved heat-sensitive flavors, yielding a pea-like odor. In contrast to prior studies limited to single methods or parameters, this work provides a comprehensive multidimensional comparison, and offers theoretical support for the high-value utilization of sea buckthorn protein hydrolysates in functional foods and nutraceuticals.

## Highlights

• Drying methods affected the structure of sea buckthorn hydrolysates.
• Vacuum freeze-dried hydrolysates show highest antioxidant capacity (DPPH/ABTS).
• Spray-dried hydrolysates had more toasted nutty; freeze-dried retained pea flavor.

## 1 Introduction

Sea buckthorn, a deciduous shrub bearing small berries, is classified within the genus *Hippophae* of the *Elaeagnaceae* family. It is notably rich in nutrients and bioactive constituents, encompassing polysaccharides, organic acids, amino acids, essential fatty acids, phytosterols, flavonoids, and mineral elements (Guo et al., 2025). Importantly, it is rich in vitamin C, ranging from 53 mg to 3909 mg/100 g. Consequently, sea buckthorn is frequently hailed as the “king of vitamin C” (Mihal et al., 2023). However, a large amount of by-products are generated during the annual processing, particularly sea buckthorn seed residue, with an annual output of 263,000 tons solely in China. This seed meal is notably nutrient-dense, particularly high in protein content. This underutilized resource can be valorized into high-value products. Transforming this underutilized resource into bioactive peptides not only helps reduce waste but also enhances economic returns, aligning with the Sustainable Development Goals (Yang et al., 2024). Similarly, jackfruit seed flour as a processing by-product has been successfully used to improve the nutritional and functional properties of pasta, showing the potential of food by-products in developing high protein and quality food products (Singh et al., 2023).

Plant proteins, a significant source of nutrition, possess high nutritional value and can be hydrolyzed into peptides via enzymatic or chemical processes. These peptides typically comprise 2–50 amino acid residues, with molecular weights ranging from 50 to 10,000 Da. These low molecular weight peptide facilitates digestion and absorption within the human body (Görgüç et al., 2020). Plant-derived bioactive compounds have attracted increasing attention due to their diverse physiological activities, including antioxidative, antibacterial, lipid-lowering, immunoregulatory, blood pressure-reducing, and blood sugar-lowering effects (Fan et al., 2025; Görgüç et al., 2020). For example, specific antioxidant peptides identified from walnut protein hydrolysates demonstrate the potential of plant-derived peptides (Chen et al., 2015). Soybean peptides offer therapeutic benefits for conditions such as cancer, oxidative stress, inflammation, immune response, and hyperlipidemia (Fan et al., 2025). Peptides derived from silver carp scales demonstrate free radical scavenging capabilities, in addition to inhibiting both tyrosinase activity and melanin production (Zu et al., 2023). Moreover, the synergistic enhancement of antioxidant and immunomodulatory activities through the combined use of non-conventional protein sources and plant extracts underscores the potential application of multi-source bioactive components in functional food development (Park et al., 2023). Sea buckthorn protein hydrolysates themselves hold great promise due to their reported biological activities, including anti-oxidation, anti-inflammatory responses, cardiovascular improvement, anti-diabetic, and anti-obesity effects (Wang et al., 2022). Although sea buckthorn protein hydrolysates exhibit broad application prospects, their industrial-scale applications in the food and pharmaceutical sectors still face multiple challenges, one of which lies in effectively maintaining structural integrity and biological activity during downstream processing (Cruz-Casas et al., 2021). Especially during the drying process, different drying technologies impose varying degrees of thermal and mechanical stress, which may induce aggregation, degradation, or conformational changes in proteins/peptides. These structural modifications can significantly alter their physicochemical properties, bioactivity, and sensory attributes, thereby limiting their application in food formulations (Wang et al., 2025). The reduction in antioxidant activity diminishes their health-promoting effects. Additionally, chemical reactions such as the Maillard reaction and lipid oxidation may occur during drying, further influencing the flavor profile of the final product. Flavor characteristics represent one of the critical factors determining consumer acceptance and commercial viability of the product (Bermúdez et al., 2014).

Although extensive research has focused on the extraction and identification of bioactive peptides from diverse sources (e.g., soybean, whey, marine organisms) and optimization of their enzymatic hydrolysis processes, systematic investigations comparing the comprehensive effects of different drying methods on the structural characteristics, antioxidant activity, and flavor profiles of specific plant-derived peptides—particularly sea buckthorn protein hydrolysates—remain relatively scarce. Most existing studies either employ a single drying method or focus exclusively on one aspect of bioactivity, failing to simultaneously evaluate the synergistic impacts of drying processes on both physicochemical properties and flavor attributes of peptides. This research gap significantly hinders the precision development and efficient utilization of sea buckthorn protein hydrolysates in functional foods and nutritional supplements.

Therefore, this study aims to systematically address the aforementioned research gaps by comprehensively comparing the effects of three commonly used drying techniques—freeze-drying, spray drying, and vacuum microwave drying—on the structural integrity, antioxidant capacity, and volatile flavor components of sea buckthorn protein hydrolysates.The specific objectives of this study include: (1) to elucidate the effects of different drying methods on the microstructure and secondary structure of sea buckthorn protein hydrolysates; (2) to evaluate the retention of antioxidant activity under three drying approaches through DPPH and ABTS radical scavenging assays as well as FRAP ferric reducing antioxidant power assay; (3) to analyze the profiles of volatile flavor compounds in sea buckthorn protein hydrolysates subjected to different drying treatments using comprehensive two-dimensional gas chromatography coupled with time-of-flight mass spectrometry (GC × GC-TOFMS).Compared to previous studies, the primary advantage of this work lies in systematically comparing the multidimensional quality changes of sea buckthorn protein hydrolysates during different drying processes. By integrating structural characteristics, antioxidant activity, and flavor attributes into a comprehensive analysis framework, this research provides critical theoretical foundations and technical support for high-quality industrial production of sea buckthorn protein hydrolysates in functional foods and nutritional supplements. These findings facilitate the effective transformation of sea buckthorn protein hydrolysates from “potential resources” to “high-value products”.

## 2 Materials and methods

### 2.1 Materials and reagents

Sea buckthorn seed protein was purchased from Peptide Love Biotechnology (Xi'an, China). Alcalase was purchased from Lonct Enzyme Preparation Co., Ltd. (Shandong, China). Trypsin was purchased from Nan Pang Bo Biological Engineering Co., Ltd. (Nanning, China). Ethanol was purchased from Aladdin (Shanghai, China). n-Dodecanol was purchased from C/D/N Isotopes Inc. (Quebec, Canada). n-Alkanes was purchased from SIGMA (USA). n-Hexane was purchased from Yonghua (Shanghai, China). 1,1-diphenyl-2-picrylhydrazyl radical scavenging activity (DPPH), and 2,2′-Azinobis-(3-ethylbenzthiazoline-6-sulphonate) (ABTS) were brought from Nanjing Jiancheng (Nanjing, China).

### 2.2 Preparation of sea buckthorn protein hydrolysates

#### 2.2.1 Enzymatic hydrolysis single factor experiment design

The optimization of enzymatic hydrolysis conditions for Alcalase was conducted, utilizing the degree of peptide hydrolysis as a performance measure. Three variables were examined in separate experiments: temperature (40 °C, 45 °C, 50 °C, 55 °C, 60 °C), pH (6, 7, 8, 9, 10), and enzyme addition (2000 U/g, 4000 U/g, 6000 U/g, 8000 U/g, 10000 U/g) (Guo et al., 2025). The initial conditions were established at 50 °C, pH 8, with an enzyme addition of 4000 U/g. The enzymatic digestion time was 1.5 h. Control variables were employed during the single-factor condition screening process.

#### 2.2.2 Enzymatic hydrolysis response surface design

Utilizing the degree of peptide hydrolysis as a criterion for evaluation in Table 1, optimal interval levels of temperature (A), pH (B), and enzyme addition (C) were determined to design a response surface experiment. The most favorable conditions for the preparation of sea buckthorn protein hydrolysates via Alcalase hydrolysis were ascertained.

**Table 1:** Experimental design matrix for response surface optimization of enzymatic hydrolysis conditions.

Factor	Level		
	-1	0	1
A T (°C)	45	50	55
B pH	9	10	11
C Amount of enzyme added (U/g)	4000	6000	8000

Note: The experimental design was based on three independent variables: temperature (A), pH (B), and Amount of enzyme added (C). Each variable was studied at three coded levels: −1 (low), 0 (central), and + 1 (high). The table lists the actual physical values associated with each coded level for all factors.

#### 2.2.3 Secondary enzymolysis

In the supernatant derived from Alcalase digestion, introduce 4000 U/g of trypsin and incubate at 37 °C under pH 8 conditions for 1.0 h. Subsequently, the final supernatant is subjected to three distinct drying techniques: vacuum freeze-drying, spray drying, and microwave vacuum drying (Yang et al., 2019).

### 2.3 Calculation of degree of hydrolysis

The pH-stat (Xiong et al., 2025) equation was used to compute the degree of hydrolysis (DH) (%) as expressed in formula (1):

(1) $$\mathrm{DH}=\frac{h}{h\mathrm{tot}}\times 100\%=\frac{V\times C}{\mathrm{Mp}\times \mathrm{hto}t\times \partial }\times 100\%$$

*Mp* is the mass of hydrolysed protein (g), *Mp* is the mass of hydrolysed protein (g), *h*_*tot*_ is the total number of peptide bonds in the protein substrate (mmol/g), and *α* is the average dissociation of *α*-NH_2_ groups. *α* is expressed as expressed in formula (2):

(2) $$\alpha =\frac{10^{\mathrm{pH}-\mathrm{pK}}}{1+10^{\mathrm{pH}-\mathrm{pK}}}$$

### 2.4 Determination of amino acid composition

The amino acid content in the sample was analyzed using high-performance liquid chromatography (Li et al., 2024). An approximate 0.2 g of the sample was precisely weighed and subsequently extracted with water followed by acetonitrile. The combined supernatants were then diluted to a total volume of 10 mL. A 200 μL portion of this extract was blended with norleucine internal standard, triethylamine acetonitrile solution, and phenylisothiocyanate acetonitrile solution. This mixture underwent derivatization at ambient temperature for an hour. Post purification with n-hexane, the concoction was filtered and introduced into the system. Chromatographic examination utilized an Athena AAA column (4.6 × 250 mm, 5 μm) where mobile phase A comprised 50 mmol/L sodium acetate (pH 6.5), and mobile phase B consisted of methanol-acetonitrile-water (20,60,20). The flow rate was maintained at 1.0 mL/min, with detection wavelength set at 254 nm and an injection volume of 10 μL. Gradient elution was executed as follows: from 0 to 39 min, the proportion of phase A reduced from 95% to 51%; between 40 and 50 min, only phase B was used; at 51 min, the original ratio was reinstated and allowed to stabilize until 60 min.

### 2.5 Morphological and structural analysis of sea buckthorn protein hydrolysates

#### 2.5.1 Scanning electron microscope image (SEM)

A suitable quantity of the sample was uniformly dispersed on the double-sided adhesive of the sample holder. Excess sample was removed by blowing before placing it in the sample chamber of the ion sputtering instrument. Following a gold spray treatment, the sample plate was transferred to the observation chamber of the scanning electron microscope for examination (Joshi et al., 2011).

#### 2.5.2 Fourier transform infrared (FTIR) spectroscopy

The potassium bromide pellet method is utilized to ensure that the sample is thoroughly ground with KBr powder in a desiccated fume hood setting. The mixture was subsequently compressed into a circular tablet, which was later subjected to examination across a spectral range of 4000–400 cm^−1^ (Xiong et al., 2025).

#### 2.5.3 X-ray diffraction analysis (XRD)

The sample was analyzed utilizing an X-ray diffraction apparatus, operated under instrument conditions of 40.0 kV and 40.0 mA. Each specimen was subjected to a continuous scan at a rate of 2°/min within the diffraction angle 2*θ*, spanning from 5° to 90° (Islam et al., 2023).

#### 2.5.4 Circular dichroism analysis (CD)

The sample was formulated into a 0.1 mg/mL aqueous solution for scanning, with a wavelength range of 192 to 240 nm. The scanning interval was set at 0.5 nm, and the circular dichroism spectrum of the sample was subsequently recorded (Yang et al., 2020).

### 2.6 Thermogravimetric analysis (TGA)

The experiment was carried out in a nitrogen atmosphere. The sample was first ground and dried, then an appropriate amount of the sample was weighed and evenly spread on the sample pan. After that, the system was purged with nitrogen gas flow to remove residual gases. During testing, programmed heating was performed at 10 °C/min while continuously recording the mass change curve of the sample as a function of temperature. An empty crucible was used as the reference baseline for the experiment (Zhu et al., 2020).

### 2.7 In vitro antioxidant activity of sea buckthorn protein hydrolysates

#### 2.7.1 DPPH radical scavenging activity

In accordance with established literature procedures (Chen et al., 2019), albeit with minor adjustments, a 0.1 mmol/L DPPH solution was formulated using anhydrous ethanol. The protein samples for analysis were prepared at concentrations of 0.5 mg/mL, 1 mg/mL, and 1.5 mg/mL in distilled water. Subsequent absorbance measurements were conducted. A mixture of 2 mL DPPH solution, and 2 mL anhydrous ethanol was used as the control group, with its absorbance A_0_ measured at 517 nm. Another mixture of 2 mL DPPH solution, and 2 mL protein sample solution was allowed to react at room temperature for 30 min in the absence of light before measuring its absorbance A_1_ at 517 nm. Additionally, a mixture of 2 mL protein sample solution, and 2 mL anhydrous ethanol was utilized as the sample blank group, and its absorbance A_2_ was also measured at 517 nm. The DPPH radical scavenging ability of the samples was subsequently computed using the appropriate formula (3).

(3) $$\mathrm{Scavenging rate of DPPH radical}\;\left(\%\right)=\left(1-\frac{A_{1}-A_{2}}{A_{0}}\right)\times 100\%$$

#### 2.7.2 ABTS radical scavenging ability

Adapting established literature procedures with minor modifications (Chen et al., 2019), 7.00 mmol/L ABTS solution was combined with a 2.45 mmol/L potassium persulfate solution in a 1:1 ratio. This mixture was allowed to react in the dark for 16 h to produce a reserve solution. When the absorbance at 734 nm of this reserve solution reached approximately 0.70 ± 0.02, it was diluted with distilled water to generate a working solution. Subsequently, 1 mL of sample solution was added to 3 mL of ABTS working solution, and the mixture was incubated in the dark for 30 min. The mixture was then centrifuged at 8000 r/min for 5 min. 200 μL of supernatant was transferred to a 96-well plate, and its absorbance value (A_1_) was measured at 734 nm. Concurrently, the absorbance values of the mixture of 1 mL sample solution with 3 mL distilled water (A_2_), and the mixture of 1 mL distilled water with 3 mL ABTS working solution (A_0_) were also determined. The ABTS radical scavenging ability of the sample was subsequently computed using the appropriate formula (4).

(4) $$\mathrm{Scavenging rate of ABTS radical}\;\left(\%\right)=\left(1-\frac{A1-A2}{A0}\right)\times 100\%$$

#### 2.7.3 Ferric ion reducing antioxidant power (FRAP)

In accordance with established literature procedures (Chen et al., 2019), with minor modifications, a working solution was prepared by mixing an acetate buffer solution (0.3 mol/L, pH 3.6), 2,4,6-Tris (2-pyridyl)-1,3,5-triazine solution (0.01 mol/L), and ferric chloride solution (0.02 mol/L) in a 10:1:1 ratio. This working solution was required to be preheated at 37 °C for a duration of 20 min prior to utilization. Subsequently, samples or distilled water were combined with 180 L of the working solution, and the reaction was constant temperature at 37 °C for 10 min, and the absorbance was measured at 593 nm. A standard curve was generated using ferrous sulfate solutions ranging from 0.003125 to 0.1 μmol/mL. This was used to calculate the FRAP value of the sample, with the regression equation being (Y = 0.1135 x + 0.1049 and R^2^ = 0.9933).

### 2.8 Comprehensive two-dimensional gas chromatography coupled with time of flight mass spectrometry (GC × GC–TOFMS)

#### 2.8.1 Internal standard solution preparation

A stock solution of 1 mg/L n-Hexyl-d13 Alcohol was prepared in 50% ethanol, while a separate 1 mg/L n-Alkanes stock solution was prepared in n-Hexane; both solutions were stored at 4 °C in a refrigerator for further use (Li et al., 2022).

#### 2.8.2 Flavor extraction

For analysis, an appropriate amount of sample was placed into a 20 mL headspace vial, followed by the addition of 10 μL of the internal standard (ISTD) solution. Separately, 10 μL of the n-Alkanes standard was transferred to another 20 mL vial for calibration. All vials were incubated at 80 °C for 10 min to equilibrate volatiles. Prior to extraction, the Solid-Phase Microextraction fiber was conditioned in the GC inlet at 270 °C for 10 min to remove contaminants. The fiber was then exposed to the sample or standard headspace at 80 °C for 25 min for analyte adsorption, followed by desorption in the GC injector at 250 °C for 5 min. After injection, the fiber was reconditioned at 270 °C for 10 min to ensure no carryover between runs (Li et al., 2022).

#### 2.8.3 The relative odor activity value analysis (ROAV)

ROAVserves as an index for evaluating the contribution of flavor substances to the overall flavor profile. A ROAV greater than 1 signifies key flavor substances making significant contributions. Conversely, a ROAV between 0.1 and 1 denotes substances that contribute to the flavor, albeit not in a prominent role. A ROAV less than 0.1 suggests a negligible contribution to the overall flavor (Xia et al., 2025).

### 2.9 Statistical analysis

The experiments were conducted in triplicate, and statistical analysis was carried out using SPSS 27. Statistical significance was determined using One-way analysis of variance (ANOVA), with significance levels set at *p* < 0.05 or *p* < 0.01.

## 3 Results and discussion

### 3.1 Experimental results of enzymatic hydrolysis process

#### 3.1.1 Results of single-factor experiments

**Fig. 1:**
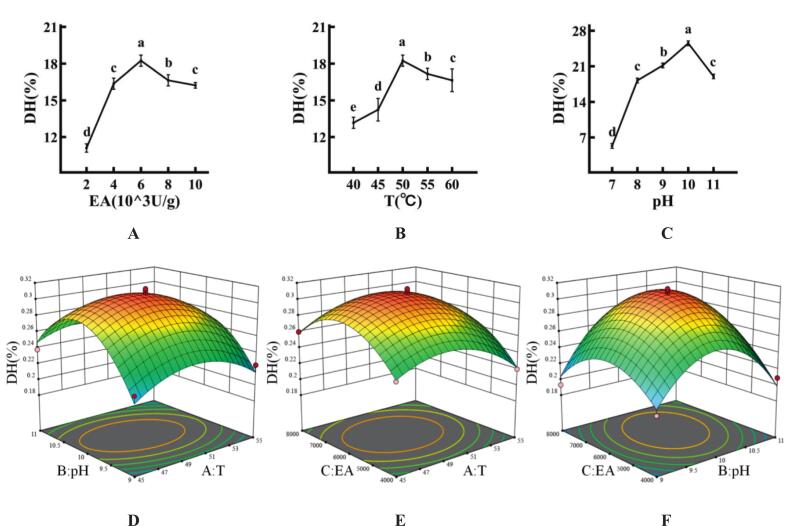
The results of single-factor and response surface experiment of sea buckthorn protein hydrolysates. (A) The influence of temperature on the hydrolysis. (B) The influence of pH on the hydrolysis. (C) The effect of enzyme addition on the hydrolysis. (D)The interaction of temperature and pH on hydrolysis. (E) The interaction of temperature and enzyme addition on hydrolysis. (F) The interaction between pH and enzyme addition on hydrolysis. Different lower case letters indicate significant differences (*p* < 0.05).

In the enzymatic hydrolysis process, the influence of temperature on the degree of hydrolysis (Fig. 1A) shows a trend of first increasing and then decreasing. The rise in temperature affected the enzymatic activity of Alcalase, which may increase the chance of the enzyme contacting the substrate, thereby accelerating the enzymatic reaction. However, excessively high temperatures can lead to the destruction of the enzyme structure, causing a reduction in the degree of hydrolysis (Accardo et al., 2022). The maximum degree of hydrolysis, 18.23 ± 0.46%, was achieved at 50 °C. Therefore, the optimal temperature chosen was 50 °C.

During the enzymatic hydrolysis process, the influence of pH on the degree of hydrolysis (Fig. 1B) shows a trend of first increasing and then decreasing. However, due to different enzymes having their optimal pH ranges, it directly affected the structure of the enzyme and its interaction with the substrate (Accardo et al., 2022). At pH 10, the degree of hydrolysis reached its maximum value of 25.53 ± 0.44%, so the optimal pH of 10 was chosen.

In the enzymatic hydrolysis process of Alcalase, the effect of enzyme addition on hydrolysis degree (Fig. 1C) shows a trend of gradually increasing and then tending to level off. Since the substrate was finite, as the amount of enzyme added increases step by step, it resulted in an excess of enzymes that did not participate in the reaction system, which in turn affected the enzymatic reaction (Hong et al., 2025). The maximum hydrolysis degree of 18.23 ± 0.44% was achieved at an enzyme dosage of 6000 U/g, thus the optimal enzyme dosage of 6000 U/g was selected.

#### 3.1.2 Results of response surface experimentation

Through a series of single-factor experiments, key parameters such as temperature, pH, and enzyme addition amount were identified for further optimization. Subsequent response surface optimization experiments were conducted to refine the enzymatic hydrolysis conditions for sea buckthorn protein hydrolysates. The Regression model and analysis of variance results were detailed in Table 2. Utilizing software for multiple regression equation fitting, Regression Equation Model (5) was obtained:

(5) $$Y=-8.66987+0.12285\;A+1.1567\mathrm{5B}+0.000035C-0.001540\mathrm{AB}+2.75\;\mathrm{AC}+6.875\;\mathrm{BCE}-0.001115A^{2}–0.055875B^{2}–9.3437{\mathrm{5C}}^{2}$$

**Table 2:** Analysis of variance for the response surface quadratic model of the degree of hydrolysis.

Souce	Sum of Squares	df	Mean Square	F-value	P-value	
Model	0.0275	9	0.0031	26.21	0.0001	**
A-T	0.0004	1	0.0004	3.86	0.0901	–
B-pH	0.0005	1	0.0005	4.39	0.0743	–
C-EA	0.0009	1	0.0009	7.57	0.0284	*
AB	0.0002	1	0.0002	1.80	0.2211	–
AC	0.0000	1	0.0000	0.2597	0.6260	–
BC	0.0008	1	0.0008	6.49	0.0382	*
A^2^	0.0033	1	0.0033	28.08	0.0011	**
B^2^	0.0131	1	0.0131	112.84	< 0.0001	**
C^2^	0.0059	1	0.0059	50.49	0.0002	**
Residual	0.0008	7	0.0001			
Lack of Fit	0.0006	3	0.0002	4.78	0.0825	–
Pure Error	0.0002	4	0.0000			

Note: The analysis of variance evaluates the significance of the model and its terms. A: Temperature, B: pH, C: Amount of enzyme added (EA). AB, AC, BC represent interaction effects, and A^2^, B^2^, C^2^ represent quadratic effects. Degrees of freedom (df). F-value and P-value indicate the statistical significance of each term.

* indicated significant difference (*P* < 0.05), ** indicated extremely significant difference (*P* < 0.01).

The results indicated that the model's *P*-value is 0.0001, and the lack of fit term was 0.0825. These values suggested that the model was highly significant and aligned closely with the actual data, thereby ensuring a reliable prediction of the degree of hydrolysis (Peng et al., 2020). Furthermore, the model's correlation coefficient R^2^ was 0.9712; a higher correlation coefficient signified a stronger correlation between the model and the data. This underscored the model's robust fitting capability, while maintaining experimental error within acceptable limits (Ruangmee & Sangwichien, 2013). The Adj-R^2^ value standed at 0.9341, highlighting that the regression equation effectively captured variations in parameters. These findings collectively attested to the model's efficacy in elucidating the process. In conclusion, utilizing this regression model for optimizing the extraction process was likely to produce commendable outcomes (Saleem et al., 2022).

Within the experimental parameters, the impact of various independent variables on the extraction rate was assessed based on the F-value. The data suggested that, when considering the differential impacted of these variables on the extraction rate, the order of influence can be ranked as: enzyme addition > pH > temperature. Notably, enzyme addition exerted the most pronounced effect on the degree of hydrolysis. The P-value for the interaction term BC was found to be <0.05, whereas the *P*-values for the interaction terms AB and AC were both >0.05. This indicated a significant interaction between pH and enzyme addition, while other interactions were deemed statistically insignificant.

#### 3.1.3 Effect of interactions on extraction rate

The effects of the interaction between various factors on the degree of hydrolysis, such as temperature and pH (Fig. 1D), temperature and enzyme addition (Fig. 1E), and enzyme addition and pH (Fig. 1F), were described respectively. The response surface diagram corresponding to the established regression model was drawn. Observations from the variations in the three-dimensional representation revealed that the amalgamation of each factor in pairs exhibited convexity, and its projection on a plane appeared as a circle. This suggested that the intensified interaction between the dependent variables leaded to an initial increase in the degree of hydrolysis followed by a decrease, indicating the emergence of an extreme value (Tahir et al., 2024).

[Fig. 1].

### 3.2 Effect of drying methods on the amino acid composition of sea buckthorn protein hydrolysates

In Table 3, the total amino acid content varied among the three drying methods, highlighting the influence of the drying process on amino acid retention (Niu et al., 2024). The total amino acid content was highest and nearly identical for microwave drying and freeze-drying, at 5.32 ± 0.09 g/100 g and 5.44 ± 0.02 g/100 g, respectively, indicating that these two methods have a stable and effective amino acid retention effect. In contrast, spray drying resulted in the lowest total amino acid content of 5.05 ± 0.28 g/100 g. This result strongly suggested that instantaneous high temperature heating during the spray drying process may cause thermal degradation or Maillard reaction of certain amino acids, resulting in loss of amino acids (Cheng et al., 2025). Freeze-drying, performed under low-temperature vacuum conditions, effectively minimizes thermal damage.

**Table 3:** Amino acid composition of sea buckthorn protein hydrolysates under three different drying methods.

Amino Acid	Drying method		
	SD-SBP(mg/g)	MVD-SBP(mg/g)	VFD-SBP(mg/g)
Asp	0.445	0.509	0.446
Glu	1.959	2.127	2.023
Ser	1.433	1.671	1.464
Gly	0.395	0.464	0.608
His	1.448	1.498	1.554
Arg	5.973	6.641	6.653
Thr	1.855	2.122	2.269
Ala	0.430	0.439	0.411
Pro	2.939	2.339	2.949
Tyr	5.405	5.862	6.016
Val	1.527	1.633	1.747
Met	0.601	0.640	0.551
Cys	0.915	0.712	0.800
IIe	2.217	2.376	2.277
Leu	9.486	9.739	9.935
Phe	7.926	8.570	8.684
Trp	1.447	1.486	1.424
Lys	4.140	4.353	4.542
EAA	30.65 ± 2.12	32.42 ± 0.51	32.98 ± 0.27
TAA	50.54 ± 2.81	53.18 ± 0.94	54.35 ± 0.21

Note:EAA: Thr, Val, Met, lle, Leu, Phe, Lys, Trp, His.

SD-SBP: Spray-dried sea buckthorn protein hydrolysates prepared.

MVD-SBP: Vacuum microwave-dried sea buckthorn protein hydrolysates prepared.

VFD-SBP: Vacuum freeze-dried sea buckthorn protein hydrolysates prepared.

The contents of individual amino acids are expressed as mean values, whereas the total and essential amino acid contents are presented as the mean ± standard deviation.

From the perspective of essential amino acids (EAA) analysis, products processed by all three drying methods showed high nutritional quality. Freeze-dried samples had the highest total EAA content (3.30 ± 0.03 g/100 g), followed by microwave-dried samples (3.24 ± 0.05 g/100 g) and spray-dried samples (3.06 ± 0.21 g/100 g). More importantly, the ratio of EAA to total amino acids was higher than 60% in all three drying methods, which was significantly higher than the FAO/WHO recommended standard for high-quality protein (40%). This suggested that the overall amino acid profile of sea buckthorn protein hydrolysates was excellent (Yu et al., 2022).

### 3.3 The impact of drying methods on the structure of sea buckthorn protein hydrolysates

#### 3.3.1 Surface microstructure

**Fig. 2:**
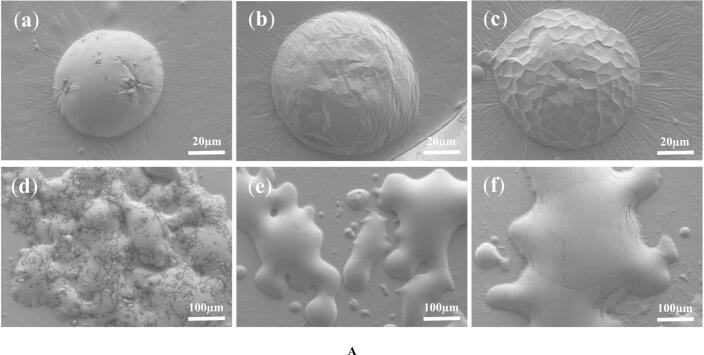

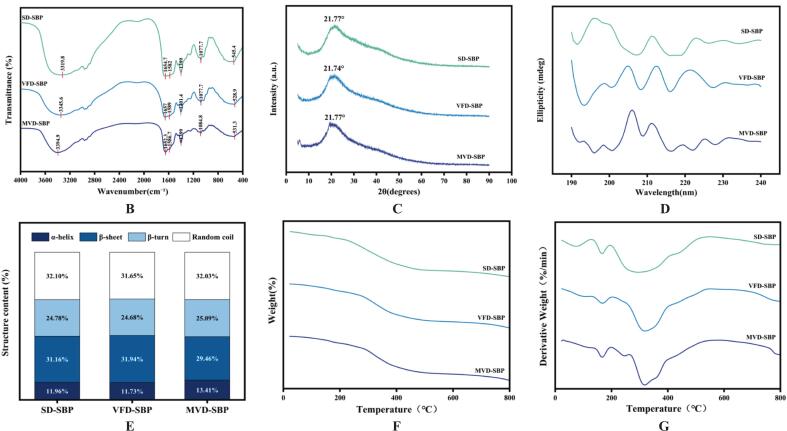
The microscopic morphology and characterization of structure of sea buckthorn protein hydrolysates from three different drying methods. A(a-c) SEM images of MVD-SBP, SD-SBP, and VFD-SBP, respectively (500×). A(d-f) SEM images of MVD-SBP, SD-SBP, and VFD-SBP, respectively (100×). (B) FTIR spectroscopy of MVD-SBP, SD-SBP, and VFD-SBP. (C) X-ray diffraction pattern of MVD-SBP, SD-SBP, and VFD-SBP. (D) Circular dichroism spectroscopy of MVD-SBP, SD-SBP, and VFD-SBP. (E) Relative content of secondary structures in circular dichroism spectroscopy results. (F) Thermogravimetric analysis of MVD-SBP, SD-SBP, and VFD-SBP. (G) Derivative thermogravimetricanalysis of MVD-SBP, SD-SBP, and VFD-SBP.

Analysis of scanning electron microscope images (Fig. 2A) revealed the morphology of MVD-SBP individual particles to be relatively spherical, albeit with minor surface damage or depressions, which were likely caused by physical damage from internal steam rapidly breaking through the incompletely solidified surface “shell.” Notably, particle agglomeration was observed, along with evidence of micro-crystallization; this may be due to the rapid and selective heating of microwave energy, which could lead to localized temperature increases sufficient to cause partial melting and subsequent recrystallization of certain small-molecular-weight peptides in the protein hydrolysate or coexisting crystallizable components, forming microcrystalline regions. Such microcrystalline structures may further influence the product's flowability and dissolution behavior. In contrast, SD-SBP employs hot air as the medium for heat exchange with the sample surface. Individual particles appeared relatively full but exhibited some surface wrinkles, a feature that may be formed during the rapid drying and shrinkage of the droplet surface during spray drying (Lin et al., 2020), reflecting the kinetic competition between crust formation and internal shrinkage, which may result in lower particle packing density and restricted flowability. VFD-SBP individual particles, on the other hand, display a honeycomb-like structure on the surface, attributable to freeze-drying, where the growth of ice crystals displaces solids into the interstices between crystals, forming the honeycomb-like pores; this highly porous structure typically corresponds to a larger specific surface area and lower bulk density. The low temperatures and sublimation process inherent in freeze-drying may also contribute to the formation of larger particles or flake-like structures (Lin et al., 2020). During freeze-drying, the resistance of the dried layer allows the structure to largely retain its pre-freezing macroscopic morphology without undergoing the intense shrinkage deformation seen in spray drying, thereby better preserving the original microstructure of the material. The observed porous structure of VFD-SBP was associated with the excellent performance of abalone bioactive peptide products. This structure was considered conducive to freeze-dried products better retaining processing performance and bioactivity (Li et al., 2025), such as faster rehydration rates and higher stability of active ingredients. However, studies have indicated that spray-dried Antarctic krill protein exhibits better physicochemical and functional properties (e.g., solubility, emulsification) than freeze-dried products. This apparent contradiction may stem from differences in materials and functional objectives (Lin et al., 2020), suggesting that the choice of drying method requires comprehensive consideration of raw material characteristics and the target product's structure and function. Simultaneously, the unique microcrystalline and agglomerated structures observed in MVD-SBP in this study are consistent with findings that combining hot air and microwave drying can also produce high-quality dried products with lower energy consumption and shorter processing times (Kaveh et al., 2021). Theae indicated that this method formed a novel structure distinct from traditional hot air or freeze-drying, potentially offering advantages in controlling product texture, release characteristics, and storage stability.

#### 3.3.2 FTIR spectroscopy analysis

FTIR spectra (Fig. 2B) serves as a valuable tool for identifying the functional groups and secondary structures of proteins. Notably, the FTIR spectra of samples exhibited variations in the absorption band spectra within the range of 4000–400 cm^−1^. An absorption peak near 3300 cm^−1^, pertaining to the amide A region, was attributed to the extension of free O—H bonds coupled with the combination of N—H bonds and hydrogen bonds (Long et al., 2024), reflecting the strength of intramolecular and intermolecular hydrogen bonds as well as the state of water molecules. Among the three analyzed samples, MVD-SBP had the highest absorption peak at 3394.9 cm^−1^, probably due to the vigorous movement and rapid evaporation of polar water molecules during microwave drying, resulting in temperature gradients that facilitated the formation of a denser hydrogen bond network between protein molecules and water molecules. This phenomenon may also indicate a relatively higher content of residual “bound water” or more uniform distribution of moisture in post-drying samples. In contrast, SD treatment might cause localized thermal stress leading to partial disruption of hydrogen bonds, while VFD processing could weaken this region's absorption because ice crystallization and sublimation partially destroyed the original hydration shell of proteins.

In the fingerprint region of 1800–1500 cm^−1^, a strong absorption peak near 1657 cm^−1^ was attributed to Amide *I* band mainly related to C = O stretching vibrations with high sensitivity to protein secondary structures (Güler et al., 2016). The relatively higher peak intensity at this position in VFD-SBP indicated that its proteins might retain more ordered secondary structure and especially higher α-helix content since the characteristic absorption of α-helix usually located in the range of 1650–1658 cm^−1^ (Güler et al., 2016). This may be due to the fact that vacuum freeze drying process could avoid exposure to high temperature and liquid water, thus minimizing thermal denaturation and hydrolysis of proteins and maintaining their natural conformation. The Amide II band near 1540 cm^−1^ was mainly caused by N—H bending and C—N stretching vibrations, while the Amide III band around 1240 cm^−1^ was primarily composed of C—N stretching and N—H bending vibrations. These two absorption peaks were observed in all samples, further confirming the existence of protein peptide bonds. In particular, the Amide II band is sensitive to hydrogen bond environment and secondary structural changes, and the shift and intensity of the peak are often associated with structural transformation such as β-sheet formation (Tian et al., 2020).

In addition, several absorption peaks in the ranges of 1400–1200 cm^−1^ and 1124–1247 cm^−1^ were attributed to C—C and C—O stretching vibrations (Xiong et al., 2025), which may be related to pyranose rings and glycosidic linkages in pectic polysaccharide components (Muhidinov et al., 2024). The spectral differences in these regions reflected the effects of different drying methods on sugar chain conformation and sugar-protein interactions. In conclusion, FTIR analysis indicated that MVD treatment enhanced hydrogen bonding between protein and water molecules in sea buckthorn protein hydrolysates, which might improve their hydration capacity and dispersibility; whereas VFD treatment maximally preserved the native secondary structure of proteins, favoring the retention of their functional properties.

#### 3.3.3 X-ray diffraction

X-ray diffraction (XRD) (Fig. 2C) is a technique that allows for the observation of changes in the crystalline structure of substances, including reflecting the formation of complexes between organic ligands and metal ions. As depicted in the figure, the MVD-SBP, SD-SBP, and VFD-SBP samples each exhibited a primary peak of low intensity and a broad base at a diffraction angle near 22°. This suggested that all three exist as irregular amorphous structures with negligible differences among them. Previous research has indicated a significant correlation between diffraction intensity and particle size. The larger protein crystal structures tended to display higher intensity. This further supported the notion that the three samples were all irregular amorphous structures with minor variances (Islam et al., 2023). However, there were still slight differences among the three. The peak of MVD-SBP around 21.77° was relatively sharper with a slightly narrower full width at half maximum, and the baseline intensity might be marginally lower than the other two samples, which perfectly corresponds to the “microcrystalline evidence” observed via SEM. The localized heating effect of microwave irradiation may induce more pronounced local rearrangement and aggregation of small molecular peptide segments, free amino acids, or coexisting sugars. Secondly, SD-SBP exhibited its characteristic peak position at approximately 21.74°, with full width at half maximum and peak intensity likely intermediate between MVD-SBP and VFD-SBP. During spray drying, rapid evaporation and surface crust formation expose molecules to elevated temperatures, providing sufficient energy for migration and accumulation albeit within an extremely short timeframe. Consequently, this process probably generates an amorphous structure with moderate compactness and medium orderliness. The “surface wrinkles” observed through SEM represent macroscopic deformation, while XRD reflects molecular-scale packing states. These complementary observations collectively characterize the features of rapid nonequilibrium drying processes. Additionally, literature reports indicated that hot-air dried samples contained the highest total amino acid content, presumably due to protein degradation or thermal decomposition generating more free amino acids. This finding corroborated our experimental results where SD-SBP demonstrated “medium orderliness” in XRD analysis and “conformational unfolding” in CD spectroscopy, jointly suggesting that thermal effected during spray drying not only altered physical structures but may also caused peptide bond cleavage or degradation, potentially modifying final product composition and flavor profiles (Yuan et al., 2025). In contrast, VFD-SBP displayed the broadest and weakest peak around 21.77°, precisely matching its SEM-revealed.

#### 3.3.4 Circular dichroism (CD)

CD is often used to analyze the secondary structure of proteins and quantify the relative content of proteins, and this study explored the effect of drying methods on the structure of peptides by CD, as shown in Fig. 2D and Fig. 2E. The far ultraviolet region (190–260) corresponded to the absorption peak range of the peptide bond, providing insightful reflections of the protein main chain conformation as well as the primary types and relative content of the secondary structure of the protein. Within this secondary structure, α-helix and β-fold are orderly structures that exhibited high stability, whereas β-turn and random coil were categorized as disordered structures (Jackson & Mantsch, 1995). As depicted in the figure, the secondary structures of VFD-SBP and MVD-SBP demonstrated basic consistency, while the characteristic peaks of SD-SBP at 208 nm and 190–200 nm have undergone changes, suggesting a decrease in the relative content of its α-helix and β-fold structures. This was most likely due to the non-equilibrium process of rapid temperature increase that samples experience during spray drying. This transient but intense thermal stress was sufficient to break down the non-covalent interactions (e.g., hydrogen bonds and hydrophobic interactions) that maintain the higher order structure of proteins/peptides, resulting in unfolding of peptide chains (Lin et al., 2020). These highly ordered α-helices and β-sheets, which are stabilized by intramolecular and intermolecular hydrogen bond networks within polypeptide chains, changed from their well organized α-helical/β-sheet conformation into a more loosely arranged disordered random coil or extended conformation. This phenomenon is consistent with the surface folding observed in SEM and the local rearrangement phenomena detected in XRD. Furthermore, VFD-SBP maximized preservation of the original conformation due to its entirely low-temperature process. In contrast, MVD-SBP benefited from microwave-induced localized heating combined with vacuum environment which lowers the boiling point, resulting in processing temperatures significantly below those of spray drying. These factors caused less disruption to peptide chain covalent bonds and weak interactions maintaining secondary structures, thereby better preserving the conformation. Consequently, both VFD-SBP and MVD-SBP exhibit superior stability compared to SD-SBP (Shevkani et al., 2019).

### 3.4 Effect of drying methods on the thermal stability of sea buckthorn protein hydrolysates

The thermal stability of the three samples was evaluated using integrated Thermogravimetric (TG) and derivative thermogravimetric (DTG) analysis as shown in Fig. 2F and Fig. 2G, which showed significant differences in their overall thermal degradation behavior. VFD-SBP and MVD-SBP had significantly higher thermal stability than SD-SBP. The initial temperature for rapid weight loss of VFD-SBP and MVD-SBP were 275.24 °C and 291.72 °C, respectively, which were much higher than that of SD-SBP (226.13 °C). Additionally, the total weight loss percentages during the main degradation stage for VFD-SBP and MVD-SBP were 43.57% and 44.96%, respectively, which were lower than that of SD-SBP (60.73%). Furthermore, the DTG curve characteristics indicated that the primary weight loss peak temperatures for VFD-SBP and MVD-SBP occurred at 319.47 °C and 317.77 °C, respectively, which were higher than that of SD-SBP (294.67 °C), with broader peaks and lower intensities, suggesting a slower decomposition rate of their components under heat exposure and relatively higher thermal stability (Hamidallah et al., 2025). In contrast, SD-SBP exhibited inferior thermal stability throughout the entire degradation process, as evidenced by its earlier onset of weight loss, greater extent of weight loss, and consistently lower degradation peak temperatures across all stages in the DTG curve with sharper peaks, indicating faster component decomposition rates and comparatively lower thermal stability (Krekora et al., 2023). It is worth noting that there was no statistically significant difference in the thermal stability between VFD-SBP and MVD-SBP, both of which demonstrated superior thermal resistance compared to SD-SBP.

[Fig. 2].

### 3.5 Analysis of antioxidant capacity

The experimental results indicated that sea buckthorn protein hydrolysates, prepared through three distinct drying methods-spray drying, microwave vacuum drying, and vacuum freeze drying all exhibited a concentration-dependent antioxidant activity in the DPPH, ABTS radical scavenging rate, and FRAP reducing power tests. Specifically, there was a notable increase in antioxidant activity corresponding to a rise in concentration, ranging from 0.5 to 1.5 mg/mL.

**Fig. 3:**
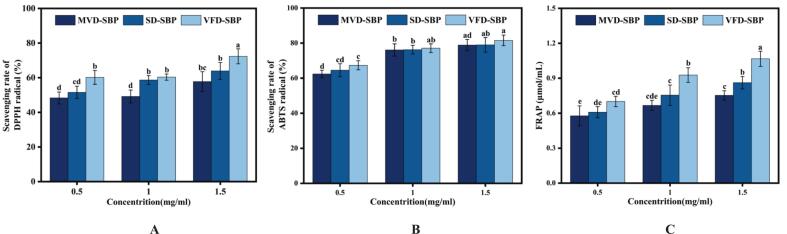
Results of antioxidant capacity of sea buckthorn protein hydrolysates prepared by three drying methods. (A) DPPH. (B) ABTS. (C) FRAP. Different lower case letters indicate significant differences (*p* < 0.05).

In the DPPH assay (Fig. 3A), VFD-SBP exhibited a clearance rate of up to 72.34 ± 4.32% at 1.5 mg/mL. This was higher than that of SD-SBP (63.89 ± 4.87%) and MVD-SBP (57.74 ± 5.69%). Similarly, in the ABTS assay (Fig. 3B), VFD-SBP achieved an 81.56 ± 2.99% clearance rate at 1.5 mg/mL, compared to 78.99 ± 4.14% for SD-SBP and 78.88 ± 3.21% for MVD-SBP. These results indicated that VFD-SBP has a strong free radical scavenging ability. In the FRAP reducing power assay (Fig. 3C), the FRAP value of VFD-SBP at a concentration of 1.5 mg/mL was notably elevated (*P* < 0.05) at 1.07 ± 0.07 μmol/mL, compared to both SD-SBP (0.86 ± 0.05 μmol/mL), and MVD-SBP (0.75 ± 0.04 μmol/mL). This finding further substantiated the superior antioxidant potential of VFD-SBP.

In the overall analysis, VFD-SBP demonstrated superior antioxidant activity across all assessments, this is in line with the view that freeze-dried chickpea protein and abalone bioactive peptides better preserve their inherent biological activities, while spray drying represents an appropriate method for maintaining the functionality of proteins (Locali-Pereira & Nicoletti, 2025). This heightened activity can likely be ascribed to the preservation of heat-sensitive active ingredients such as polyphenols and flavonoids due to its low-temperature, low-pressure processing methodology (Ma & Sam, 2024). Conversely, SD-SBP may exhibit slightly diminished activity, attributable to the degradation of certain components induced by elevated temperatures (Ma et al., 2023). MVD-SBP, while exhibiting the weakest antioxidant impact in comparison to other methodologies, demonstrated a relatively minor difference from SD-SBP. Based on the comprehensive analysis of the experimental results of structural features and antioxidant activity, VFD-SBP and SD-SBP with higher antioxidant activity were selected for comparison of sensory flavor characteristics.

### 3.6 Comparison of the sensory flavor characteristics of VFD-SBP and SD-SBP

**Fig. 4:**
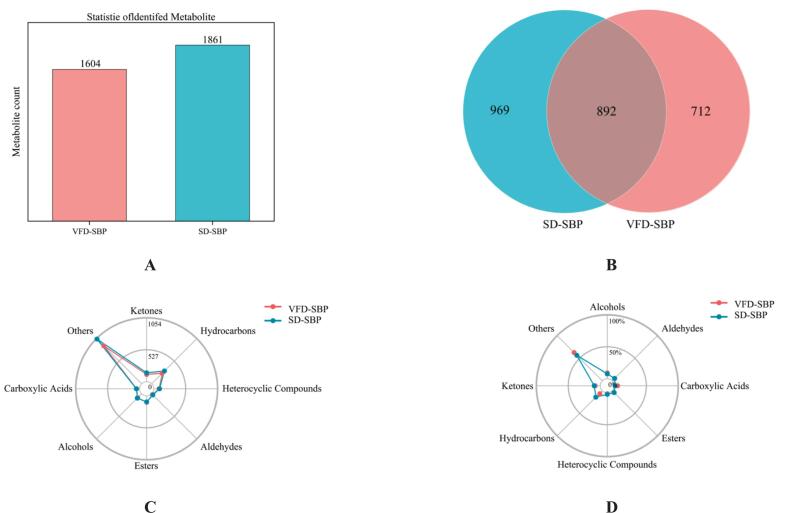
The number of substance identifications and their classified content diagram for VFD-SBP and SD-SBP. (A) Bar chart of the number of substances identified in VFD-SBP and SD-SBP. (B) Venn diagram of the number of overlapping substances in VFD-SBP and SD-SBP. (C) RadaVFD-SBP and SD-SBP. (D) Radar chart of material number and relative content of VFD-SBP and SD-SBP.

Comprehensive two-dimensional gas chromatography-time of flight mass spectrometry is aptly suited for the analysis of gases and volatile substances. Fig. 4A analytical results demonstrated that 1604 flavoring substances were identified in VFD-SBP, compared to 1861 in SD-SBP. Fig. 4B showed that there were 892 kinds of common flavoring substances in the two drying methods, which probably constituted the core flavor components of sea buckthorn protein hydrolysates. Upon further analysis, it was observed that the total number of flavoring substances in SD-SBP was approximately 16% higher than in VFD-SBP. This discrepancy indicated that the spray drying process may have superior retention efficiency or transformation generation of flavoring substances (Dutta et al., 2018).

#### 3.6.1 Volatile substance types analysis

Fig. 4C results showed that sea buckthorn protein hydrolysates samples possessed a rich and diverse array of flavor substances, primarily comprising hydrocarbons, aldehydes, esters, acids, ketones, alcohols, ethers, phenols, and heterocyclic compounds, among other volatile components. A comparative analysis suggested that SD-SBP was notably superior in retaining and generating esters, heterocyclic compounds, hydrocarbons, and ketones. In contrast, VFD-SBP exhibited a clear edge in preserving alcohols, carboxylic acids, and other heat-sensitive substances. Detailed numerical analysis and radar chart (Fig. 4D) visualizations confirmed that the relative content of alcohols, carboxylic acids, and other flavor constituents in VFD-SBP surpassed that in SD-SBP by approximately 23.5%, 18.7%, and 15.2%, respectively. This disparity implied that the vacuum freeze-drying process may better preserve heat-sensitive or highly volatile flavor compounds (Ma et al., 2024). This may confer a more pronounced sweet taste and a complex flavor signature to the sea buckthorn protein hydrolysates. Conversely, the relative concentrations of esters, heterocyclic compounds, hydrocarbons, and ketones were markedly higher in SD-SBP, exceeding those in VFD-SBP by roughly 31.2%, 26.8%, 19.4%, and 12.3% respectively. The results suggested that the spray drying process might confer a richer floral, roasted aroma and fruity nuance to sea buckthorn protein hydrolysates (Ma et al., 2024).

[Fig. 4].

#### 3.6.2 OPLS-DA of volatile metabolites

**Fig. 5:**
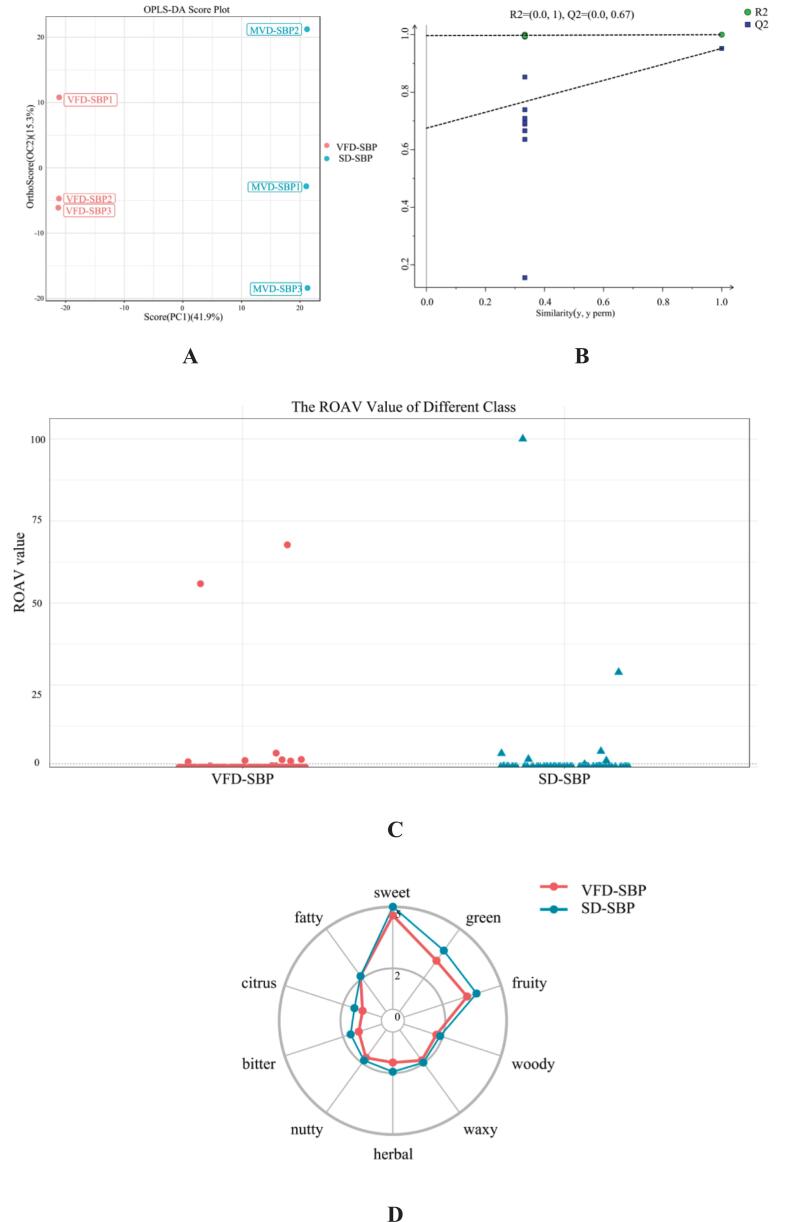
Analysis of sensory characteristics and flavor of VFD-SBP and SD-SBP. (A) OPLS-DA score plot of VFD-SBP and SD-SBP. (B) PLS-DA permutation test of VFD-SBP and SD-SBP. (C) Scatter plot of odor activity values for VFD-SBP and SD-SBP ROAV. (D) Radar chart of sensory flavor profile analysis of VFD-SBP and SD-SBP.

The utilization of the orthogonal projections to latent structures discriminant analysis (OPLS-DA) (Fig. 5A) method effectively eliminates both the within-group error and random error associated with the volatile substance content in the sample, thereby facilitating a more accurate comparison between groups. As depicted in the figure, the PC1 scores for the two drying methods exhibited obvious differences in both positive and negative modes. This suggested significant disparities in the volatile content between the two groups. Following 100 permutation tests (Fig. 5B), the interpretation rate R2 is observed to be 1.0, while the prediction rate Q2 is 0.67. Notably, all Q2 points from left to right are positioned below the original Q2 point on the far right. This indicated that the OPLS-DA method provided a better explanation for the observed differences in the volatile content of sea buckthorn protein hydrolysates samples subjected to the two distinct drying methods (Kong et al., 2015).

#### 3.6.3 Key flavor compounds and their flavor characteristics analysis

As shown (Fig. 5C) the key flavor compounds with the highest ROAV values in both drying methods were 2-Pentylfuran and 2,3,5-trimethylpyrazine, with ROAVs of 55.91 and 67.72 for SD-SBP and 100 and 28.91 for VFD-SBP, respectively. A comprehensive list of the key aroma-active compounds, including their flavor characteristics, Variable Importance in Projection (VIP) scores from the OPLS-DA model, log₂ fold change (log₂FC), and detailed ROAVs for both samples, is provided in Table 4.This suggested that 2-pentylfuran contributed more significantly to the VFD-SBP, potentially imparting a stronger green bean or vegetable flavor (Xia et al., 2025).

**Table 4:** Key discriminant aroma-active compounds and their odor activity in VFD-SBP and SD-SBP.

Compound	Class	CAS	Flavor Characteristics	VIP	log_2_FC	ROAV (VFD-SBP)	ROAV (SD-SBP)
2-Pentylfuran	Organoheterocyclic compounds	3777-69-3	Green Beans, Vegetable，fruity, beany, metallic, earthy, butter	1.531	1.47	55.91	100.00
2,3,5-Trimethylpyrazine	Organoheterocyclic compounds	14,667–55-1	–	1.503	−1.81	28.91	67.72
Acetaldehyde	Aldehydes	75–07-0	pungent, fruity, fresh, green	1.404	−0.43	1.87	0.90
Methyleugenol	Phenolic ethers	93–15-2	coconut, apricot, creamy, anise, vanilla, chocolate, mesquite, cinnamon	1.485	1.42	–	–
(*E*)-beta-Farnesene	Terpenes	18,794–84-8	–	1.544	9.04	–	–
Linalool	Lipids and lipid-like molecules	78–70-6	flower, lavender, orange, floral, sweet, lemon, blueberry, citrus	1.520	−1.53	0.0042	0.00096
Pyrazine	Organoheterocyclic compounds	290–37-9	hazelnut, pungent, barley, roasted, sweet corn	1.537	2.57	–	–
2,4-Di-tert-butylphenol	Benzenoids	96–76-4	–	1.397	0.85	–	–
Indole	Organoheterocyclic compounds	120–72–9	honey, jasmine, fishy, naphthelene, burnt, floral,	1.478	−1.44	–	–
Decanal	Aldehydes	112–31-2	floral, sweet, citrus, soap, orange peel, waxy	1.491	7.34	–	–

Notes: VFD-SBP: Vacuum freeze-dried sea buckthorn protein hydrolysates prepared; SD-SBP: Spray-dried sea buckthorn protein hydrolysates prepared.

VIP: Variable Importance in Projection from the OPLS-DA model. Values >1.0 are considered significant contributors to group discrimination.

log₂FC: log₂ (Fold Change) of compound concentration (VFD-SBP / SD-SBP). Positive values indicate higher abundance in VFD-SBP.

ROAV: Relative Odor Activity Value. Compounds with ROAV ≥1 are considered key contributors to the overall aroma. Values between 0.1 and 1 are considered modifiers.

“-”: Indicates that the compound was either not detected, or its calculated ROAV was <1, and thus it was not classified as a key aroma-active compound in that specific sample.

The ROAV of acetaldehyde was higher in VFD-SBP (1.87 vs 0.90), indicating a greater contribution to the fruity or grassy aroma in freeze-dried samples (Hidalgo & Zamora, 2019). The 2,3,5-trimethylpyrazine contributed more to the SD-SBP, possibly endowing it with a more pronounced roasted nut or cocoa flavor (Liang et al., 2022). Furthermore, The analysis results combined with Fig. 5D show, SD-SBP had greater contributions from key flavor compounds in aldehydes, ketones, and heterocyclic compounds, potentially enriching the fruity, roasted nut, and cocoa flavors. Conversely, VFD-SBP showed a significantly higher contribution from 2-pentylfuran, potentially enhancing the green bean or vegetable flavor. Other notable compounds identified in Table 4 further delineate the distinct flavor pathways induced by the different drying processes. Each drying method had its unique advantages, and the choice depends on the desired flavor profile.

[Fig. 5].

#### 3.6.4 Differences in volatile content analysis

**Fig. 6:**
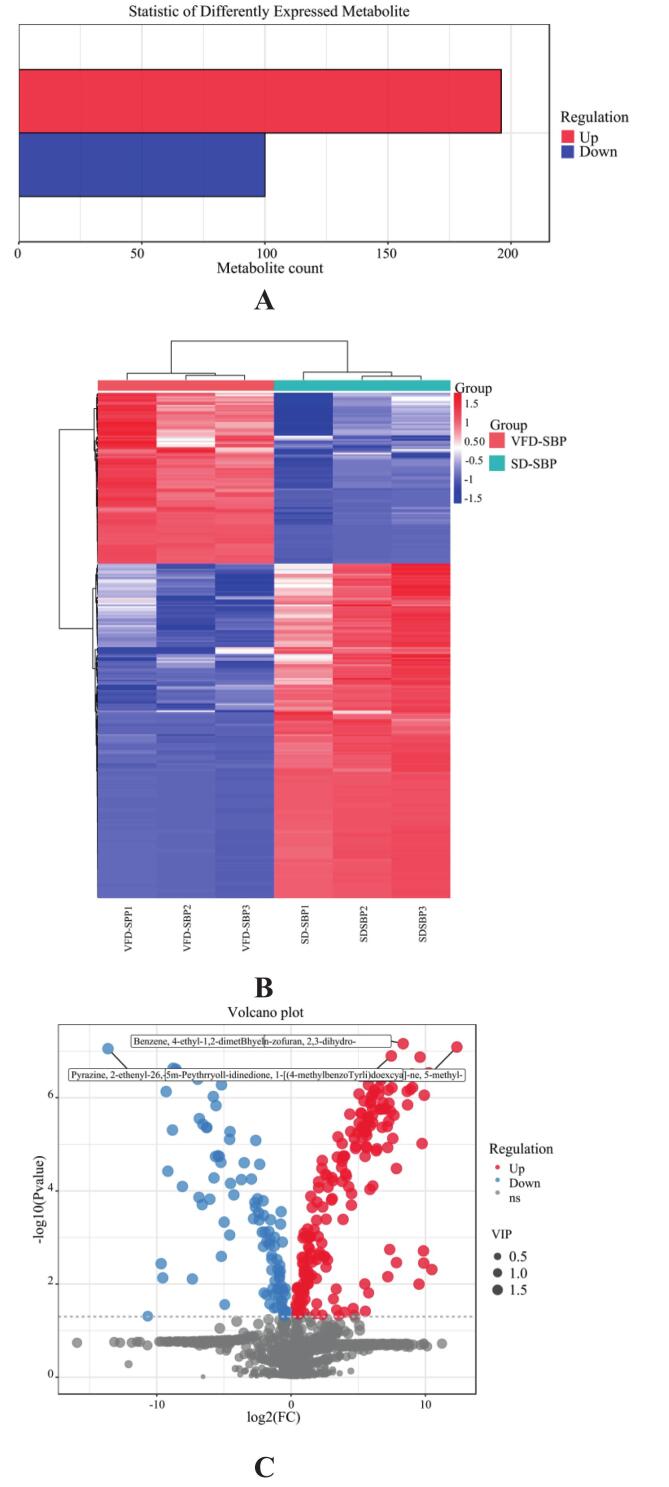

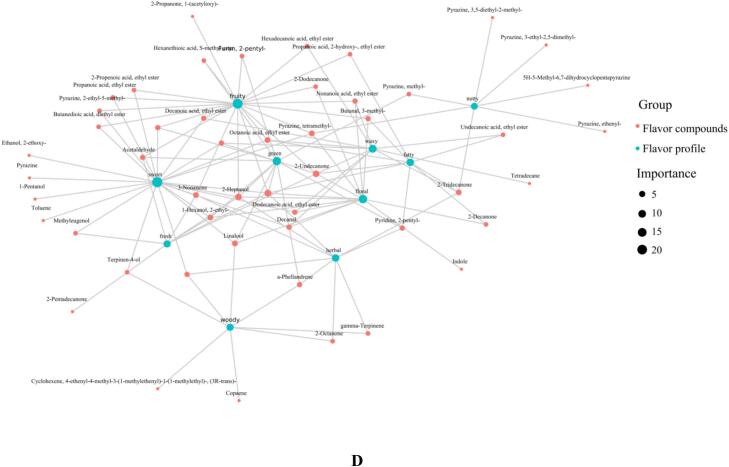
Analysis of differential substances in VFD-SBP and SD-SBP. (A) Number of differential substances between VFD-SBP and SD-SBP. (B) Heat map of differential molecules in VFD-SBP and SD-SBP. (C) Volcano plot of differential substances in VFD-SBP and SD-SBP. (D) The sensory flavor characteristics of VFD-SBP and SD-SBP associated with the flavor substance network diagram.

In comparing VFD-SBP and SD-SBP, a total of 1285 metabolites were identified. Of these differential metabolites, 196 demonstrated significantly higher expression in VFD-SBP compared to SD-SBP, while 100 displayed greater expression in SD-SBP compared to VFD-SBP (Fig. 6A). After comparing the sample difference molecular heat map (Fig. 6B) and the sample difference volcano plot (Fig. 6C) analysis, it was concluded that, it was determined that VFD-SBP notably outperformed SD-SBP in heat-sensitive substances (such as (*E*)-beta-Farnesene, 1-Penten-3-ol), volatile substances (such as 2,4-Di-tert-butylphenol), and antioxidant substances (such as 2,6-Di-tert-butyl-4-hydroxy-4-methylcyclohexa-2,5-dien-1-one). Under high temperature drying conditions, the heat-sensitive substances and volatile substances of sea buckthorn protein hydrolysates were more easily lost (Ma & Sam, 2024).

#### 3.6.5 Sensory analysis of flavor components and their correlation network diagram

The complex aroma of sea buckthorn protein hydrolysates was examined in this study, with sensory flavor characteristics identified by comparing the obtained data with the Flavor DB database. A correlation network diagram (Fig. 6D) for flavor compounds in sea buckthorn protein hydrolysates was constructed to enhance understanding of the substances contributing to the flavor. The graph generated from the sensory flavor characteristics and related flavor substances indicated that VFD-SBP may retain more volatile flavor substances such as 1-Hexanol, 2-ethyl- and Linalool. These compounds were typically associated with fresh floral, fruity and other sensory attributes. Conversely, SD-SBP exhibited stronger spice and other sensory characteristics due to substances like Methyleugenol (Guo et al., 2024). These compounds, along with key flavor substances, collectively contributed to the complex aroma of sea buckthorn protein hydrolysates.

[Fig. 6].

## 4 Conclusion

This study systematically evaluated the impact of three drying methods—vacuum freeze-drying (VFD), spray drying (SD), and microwave vacuum drying (MVD)—on the structure, antioxidant activity, and flavor profile of sea buckthorn protein hydrolysates (SBP). The objective was to provide a theoretical and technical basis for the industrial production of high-quality functional foods and nutritional supplements. Initially, the optimal conditions for Alcalase enzymatic hydrolysis in SBP preparation were determined. Structural characterization revealed that VFD-SBP more effectively maintained the inherent secondary structure of proteins, exhibiting significantly higher stability than both SD-SBD and MVD-SBP. Thermogravimetric analysis showed that both VFD-SBP and MVD-SBP had superior thermal stability. In terms of antioxidant activity, VFD-SBP displayed the most potent free radical scavenging capacity and iron-reducing ability, markedly outperforming SD-SBP and MVD-SBP. Flavor analysis indicated that SD-SBP contained a wider range of volatile compounds, resulting in a stronger roasted nutty and fruity aroma compared to VFD-SBP. In contrast, VFD-SBP was superior in retaining heat-sensitive flavor compounds, thereby more distinctly capturing green bean or vegetable-like flavors. Sensory flavor network analysis further demonstrated that VFD-SBP retained more volatile components associated with floral and fruity notes, while SD-SBP amplified spicy and other flavor attributes. This research aims to lay a comprehensive foundation for the industrial production of high-quality functional foods and nutritional supplements.

## 5 Future research directions

Although this study revealed the effects of different drying methods on the macroscopic properties of sea buckthorn protein hydrolysates, there are still many underlying mechanisms and application directions to be explored. In future studies, molecular dynamics simulation combined with peptidomics and targeted metabolomics can be used to clarify the specific transformation pathways of key peptide sequences, intermolecular interactions, and intermediate products of Maillard reaction during the drying process, so as to explain the evolution mechanism of structure–function relationship at the molecular level. Secondly, since this study only focused on the antioxidant activity in vitro, cell models (such as oxidative stress model) or animal experiments could be used to verify the actual antioxidant and anti-inflammatory effects of sea buckthorn protein hydrolysates obtained by different drying methods in biological systems, and explore their digestion/absorption potential and gut microbiota regulation ability. Moreover, optimized drying processes could be applied to prepare sea buckthorn protein hydrolysates for adding into specific food systems, systematically evaluating their processing suitability, storage stability, and sensory quality and nutritional quality of final products. Through these in-depth studies, it is expected that sea buckthorn protein resources will achieve precise conversion from “potential raw materials” to “customized high value-added functional ingredients”.

## Author contribution

Chen Chen: Executed the studies, analyzed experimental data, and wrote manuscripts. All the authors: Read and checked the manuscript.

## CRediT authorship contribution statement

**Chen Chen:** Writing – review & editing, Writing – original draft. **Pan Zhang:** Writing – review & editing. **Yuqian Wei:** Writing – review & editing. **Xiaoyue Liang:** Writing – review & editing. **Le Wang:** Writing – review & editing. **Xizhe Fu:** Writing – review & editing. **Jian Zhang:** Writing – review & editing. **Yue Zhao:** Writing – review & editing, Funding acquisition.

## Declaration of competing interest

The authors declare that they have no known competing financial interests or personal relationships that could have appeared to influence the work reported in this paper.

## Data Availability

Data will be made available on request.
